# Supplementary material for: From sequence to enzyme mechanism using multi-label machine learning
Source: BMC Bioinformatics. 2014 May 19;15:150. doi: 10.1186/1471-2105-15-150 (PMC4229970; doi:10.1186/1471-2105-15-150)
Supplement: Additional file 2 — Java code of ml2db. Additional file ml2db_code.tar.gz contains the Java source code to run the multi-label machine learning experiments and save the results to database. The code’s Javadoc is included. [file 1471-2105-15-150-S2.zip › additional file 2/ml2db/utils/doc/uk/ac/ed/inf/utils/package-tree.html]

uk.ac.ed.inf.utils Class Hierarchy


---


|  |  |  |
| --- | --- | --- |
| **Overview** \| **Package** \| Class \| Use \| **Tree** \| **Deprecated** \| **Index** \| **Help** | |  |
| **PREV**   **NEXT** | **FRAMES**    **NO FRAMES**     **All Classes** |  |


---


## Hierarchy For Package uk.ac.ed.inf.utils

**Package Hierarchies:**: All Packages

---

## Class Hierarchy

- java.lang.Object
  - uk.ac.ed.inf.utils.**EntrezUtils**- uk.ac.ed.inf.utils.**FileUtils**- uk.ac.ed.inf.utils.**Initialised** (implements uk.ac.ed.inf.utils.Initialisable)- uk.ac.ed.inf.utils.**LoggerCreator**- uk.ac.ed.inf.utils.**LogUtils**- uk.ac.ed.inf.utils.**NumberUtils**- uk.ac.ed.inf.utils.**PathUtils**- uk.ac.ed.inf.utils.**PropertiesUtils**- uk.ac.ed.inf.utils.**ReflectionUtils**- uk.ac.ed.inf.utils.**RegExpUtils**- uk.ac.ed.inf.utils.**TimeUtils**- uk.ac.ed.inf.utils.**Utils**

## Interface Hierarchy

- uk.ac.ed.inf.utils.**Initialisable**

---


|  |  |  |
| --- | --- | --- |
| **Overview** \| **Package** \| Class \| Use \| **Tree** \| **Deprecated** \| **Index** \| **Help** | |  |
| **PREV**   **NEXT** | **FRAMES**    **NO FRAMES**     **All Classes** |  |


---
